# A Tabu Search WSN Deployment Method for Monitoring Geographically Irregular Distributed Events

**DOI:** 10.3390/s90301625

**Published:** 2009-03-09

**Authors:** Nadjib Aitsaadi, Nadjib Achir, Khaled Boussetta, Guy Pujolle

**Affiliations:** 1 LIP6, University Paris 6, 104 avenue du president Kennedy, 75016 Paris, France; 2 L2TI, University Paris 13, 99 Avenue J-B Clément, 93430 Villetaneuse, France

**Keywords:** Wireless Sensor Network Deployment, Differentiated detection, Connectivity, Tabu Search heuristic

## Abstract

In this paper, we address the Wireless Sensor Network (WSN) deployment issue. We assume that the observed area is characterized by the geographical irregularity of the sensed events. Formally, we consider that each point in the deployment area is associated a differentiated detection probability threshold, which must be satisfied by our deployment method. Our resulting WSN deployment problem is formulated as a Multi-Objectives Optimization problem, which seeks to reduce the gap between the generated events detection probabilities and the required thresholds while minimizing the number of deployed sensors. To overcome the computational complexity of an exact resolution, we propose an original pseudo-random approach based on the Tabu Search heuristic. Simulations show that our proposal achieves better performances than several other approaches proposed in the literature. In the last part of this paper, we generalize the deployment problem by including the wireless communication network connectivity constraint. Thus, we extend our proposal to ensure that the resulting WSN topology is connected even if a sensor communication range takes small values.

## 1. Introduction

Over the last decade, Wireless Sensor Networks (WSN) have generated a considerable enthusiasm from the networking researchers community. Many efforts have been produced in order to apply WSN to a wide range of applications, such as: environmental monitoring, military target tracking, weather forecast, home automation, intrusion detection, etc. Basically, a WSN is a collection of small resource-constrained devices, which are typically composed of sensing components, computer processor, memory chips, and radio interface for transmitting and receiving information. The sensors could collaborate in order to observe and report the events occurring in their environment. When an event is detected, this information is routed from one node to another (multi-hops communication) and eventually gathered in gateway nodes or base stations.

While the set of challenges in wireless sensor networks are diverse, researchers have mainly focused their efforts on fundamental networking challenges, which include: routing protocols, energy minimization, sensor localization, data gathering, etc [1]. In this paper we address a static wireless sensor network deployment problem. The performances of proposed solutions related to the protocol stack depend strongly on the network deployment process. The latter one consists in determining the required number of sensors and their positions to satisfy a certain number of constraints. Classical constraints are: coverage, events reliable detection, connectivity, etc.

In this paper, we relax some of the assumptions that were considered in the literature with the aim to formulate a more realistic WSN deployment problem. The first generalization concerns the detection model. Indeed, most of works regarding WSN deployment assume a *binary detection model*. In this paper, a sensor is supposed to surely detect (detection probability is equal to 1) an event if and only if the distance between the sensor and the event is less than a particular sensing range. This model was mainly considered in works addressing area coverage problems, such as target detection or *k*-coverage problem [2]. The binary model intends to simplify the problem formulation and resolution. Unfortunately, it is not realistic, since the detection of an event depends on multiples factors, including: the distance between the sources and the sensor, the propagation signal attenuation and the accuracy of the sensing level. We believe that a distance-based probabilistic detection model, including the sensor’s technology and the event characteristics would be more realistic.

The second assumption, which was generally considered in the published papers, deals with the fact that the sensing requirement is uniformly distributed within the area. In other words, all the points of the area under monotoring are considered with the same importance. We believe that this assumption does not hold in many environments. Indeed, in many sensor network applications (such as fire detection alarms, water quality monitoring, etc.), the supervised area can request different detection levels, depending on the event’s location. For example, in the case of a fire detection system, high detection probabilities (close to 1) can be required for risky areas (example, those close to chemical deposits or to habitats). However, for low fire risky places a relative low detection probabilities are sufficient.

The third assumption consists on the wireless sensor communication range. Indeed, some existing works suppose that the communication range is very large. Consequently once the sensors are deployed, the resulting wireless communication network graph is supposed to be connected. Clearly, this assumption is not realistic in WSN. Besides, very large transmission range is not adapted in WSN as it implies a great energy consumption. As a matter of fact, in order to maximize the network lifetime, many works [3] propose solutions which aim to reduce the wireless sensor communication range. In our case study, we assume a reasonable fixed wireless sensor communication range, noted *R_c_*. Our new proposal aims to guarantee the wireless communication network connectivity. To satisfy this new constraint, our proposed method must achieve that the maximum distance separating a sensor with one of its neighbors must be less than *R_c_*.

We claim that a differentiated WSN deployment strategy, which takes into consideration the geographical characteristics of the monitored events and the wireless communication network connectivity, is more suitable in a realistic case study. Therefore, in this paper, we address the issue of deploying WSN by ensuring the network connectivity and the deployment field characterized by: (*1*) a probabilistic event detection model and (*2*) a geographical irregularity of the sensed event. To solve this problem we propose an original pseudo-random method, based on the Tabu Search algorithm.

The rest of this paper is structured as follows: the next section provides a state of the art on WSN deployment issue. The generalized problem is formalized in section 3.. Our Tabu Search-based Differentiated Deployment approach is detailed in section 4.. Performance evaluations are then discussed in section 5.. Finally section 6. summarizes our contributions.

## 2. Related Works

In the recent years, there have been many research activities and advances in sensor networks. However, a small number of them have addressed WSN deployment process. In the literature, we found some works which studied the deployment with a probabilistic detection model. Other works integrate the connectivity constraints but assume a binary detection model. To the best of our knowledge, no work have yet considered the deployment process including at the same time the network connectivity requirement, a probabilistic detection model, and a non-uniform event detection probabilities constraints.

Earlier works such as [4–6] considered that the environment is unknown or that the area is inaccessible (e.g. a battle field). Consequently, random geographical sensors deployment is assumed. On the other hand, some works considered that sensors are likely to be deployed in a regular structured manner through hand placement (e.g. based on grid). Such approach is adapted to monitor a phenomenon with a sensing characteristic that is uniformly distributed in an area.

Krishnendu and al. [7] used a binary detection model and presented a grid coverage strategy for effective surveillance and target location in distributed WSN. They have considered two types of sensors having with different characteristics (cost and range). Thereafter, they have considered an integer linear programming (ILP) approach to find the solution which minimizes the sensors’s cost while ensuring the complete coverage of the studied area. Note that, although the ILP could lead to obtain exact resolution, this method will raise important computational complexity for realistic case studies (especially for large surface areas), since the problem under study is known to be NP-hard.

In [8], the authors assumed a probabilistic detection model, which is expressed as an exponential function of the distance between the location of the targeted event and the sensor position. This model was considered in the proposal of two new deployment algorithms. Both approaches seek toward an optimal area coverage under the constraints of imprecise detections. The solutions are based on a grid structure. The first algorithm, *Max_Avg_Cov*, aims to maximize the average coverage of the grid points. The second one, *Max_Min_Cov* aims to maximize the coverage of the grid points which are the least effectively covered. Both *Max_Avg_Cov* and *Max_Min_Cov* were initially designed for an area with uniform detection probability, however they can easily adapted in the case of an area with non-uniform detection probability. Unfortunately, the two strategies suffer from high computational complexity, it is equal to *O*(*n*^4^).

In [9], the authors proposed a new deployment strategy called *Min-Miss*. This strategy is an iterative algorithm, one sensor is deployed at each step. The authors defined for each point, in the deployment field, a new metric named over miss probability. The later quantifies the benefit in coverage when a new sensor is added. The main idea of *Min-Miss* is the following. At first, all possible free grid points (no sensors are deployed in these points) are selected to form a set. Thereafter, for each point of this set the authors compute the over miss probability metric. Finally, a new sensor is deployed at point that minimizes the over miss probability metric. That means, a sensor is deployed in a position that maximizes the event detection probabilities in the deployment field. The main drawback of this approach is the huge computational complexity of such operation, which is equal to *O*(*n*^6^).

In [10], the authors addressed explicitly a WSN deployment problem for non-uniform detection requirement. As in [8], they propose to use a probabilistic detections model. They formulated the differentiated deployment problem as an integer linear programming deployment problem, which has been proved to be NP-hard. Finally, they propose a deployment heuristics, named *Diff_Deploy*. As in [9], the main drawback of this solution is the computational complexity which is equal to $O\left(\frac{4}{3}n^{6}\right)$.

In [11], the authors proposed the Differentiated Deployment Algorithm (*DDA*), which is inspired from image processing and 3D modeling, namely mesh representation. Generally, mesh representation allows convenient modeling of arbitrary surfaces, where meshes serve as basic primitives to approximate a surface. For sensor deployment, the authors used an unstructured triangle mesh representation of the target area. Basically, the meshes nodes represent sensor positions and each arc is the Euclidean distance between the sensors. The main idea of algorithm is to permit meshes division as long as the required detection probability is not reached or the mesh division is not possible. During the mesh division process, each mesh division consists to deploy a new sensor. Unfortunately, the *DDA* suffers form high computational complexity, equal to *O*(*n*^6^).

In [12] and [13], the authors derive a sufficient conditions to guarantee a full coverage and the wireless communication network connectivity. The conditions are dependent on the communication and sensing ranges. For example in [12], the authors prove that if $R_{c}\geq \sqrt{3}R_{\max}$ (where *R_max_* is the sensing range) and the area is full covered then the wireless communication network graph is connected. The authors have even considered the *K*-connectivity case. Unfortunately a binary event detection model was supposed in this work.

## 3. Problem Statement

### 3.1. Distance-related Probabilistic Sensor Detection Model

**Figure 1.**
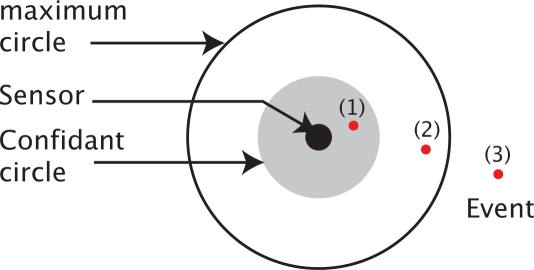
Sensor detection model

Rather than the binary detection model, and in a seek for more realism, we consider as in [14] and [15] a Probabilistic Sensor Detection Model. More precisely, we assume that event detection ability of a sensor diminishes as its distance to the sensed point increases. Using this model, a *confidant* and *maximum* sensor monitoring circles are defined. If any event (event *(1)* in Fig. 1) occurs within the *confident* circle, then event detection probability is considered as equal to 1. If the event (event *(2)* in Fig. 1) occurs outside the *confidant* circle but within the *maximum* circle, then the detection probability decreases with the distance. Finally, when the distance is larger than the radius of the *maximum* circle, then the event (event *(3)* in Fig. 1) is no longer detected. Based on the above assumption, we consider, as in [16], the following expression of the general detection probability *P* of a sensor *s* at an arbitrary point *p*:

(1) $$P(s,\;p)=\{\begin{array}{cc} 1 & \text{if}\left\|\mathrm{sp}\right\|≼1\\ \frac{\alpha }{{\left\|\mathrm{sp}\right\|}^{\beta }} & \text{if}\;1≺\left\|\mathrm{sp}\right\|≼R_{\max}\\ 0 & \text{if}\;R_{\text{max}}≺\left\|\mathrm{sp}\right\| \end{array}$$

where *R_max_* is the radius of the *maximum* circle, *α* is a sensor technological related parameter and *β* is an event characteristic-dependant parameter. Finally, ‖*sp*‖ is the Euclidean distance between the sensor *s* and the point *p*. We believe that this model is more realistic than the binary model. Therefore, we assume it works with this new model for our deployment problem, which we formulated in the next section.

### 3.2. Problem Formalization

We consider a sensor field area, denoted *𝒜*. In order to reduce the computational complexity of the problem, the area is discretized. We suppose that *𝒜* is a square, with a side equals to *n* units. A unit is defined as a normalized physical distance (example, 1 meter or 10 meters). To simplify, we will refer in the rest of this paper, to each square unit of *𝒜* by its barycenter **point**. In other words, when we say that a sensor is located in the point *p*_(*i,j*)_ *∈ 𝒜* then this means that the sensor is placed in the barycenter of the corresponding square unit. Similarly, the event detection probability of a unit square is computed considering the event detection probability of its barycenter. Finally, we consider that any event occurring inside a unit square is surely detected (event probability equals to 1) by a sensor which would be placed in its barycenter point.

In an efficiently monitoring, the event detection is formalized as a probabilistic detection model. Each point *p*_(*i,j*)_ in *𝒜* is associated a required minimum probability detection threshold, denoted *r*_(*i,j*)_.

Ideally, a successful WSN deployment algorithm should lead to obtain that ∀*p*_(*i,j*)_ *∈ 𝒜* the measured detection probability of that point is greater than *r*_(*i,j*)_, and the sensor graph of connectivity *G* is connected. The detection probability in a point *p*_(*i,j*)_ is estimated by all the sensors in its vicinity, but the event detection model is not collaborative. The detection probability of the point *p*_(*i,j*)_, denoted *𝒫*_(*i,j*)_, is estimated by all the sensors available in the monitored area as

(2) $${𝒫}_{\left(i,j\right)}=1-\prod_{(x,y)\in \mathrm{Grid}}{\left[1-P((i,j),(x,y))\right]}^{D(x,y)}$$

where *D*(*x, y*) denotes the deployment bivalent variable. If *D*(*x, y*) equals to 0 means that no sensor is deployed at grid point *p*(*i, j*). If *D*(*x, y*) equals to 1 means that a sensor is deployed at grid point *p*(*x, y*).

Obviously, if a sufficiently large number of sensors are deployed, it is possible to satisfy the objective: *𝒫*_(*i,j*)_ ≥ *r*_(*i,j*)_, ∀*p*_(*i,j*)_ ∈ *𝒫*. Nevertheless, taking into account cost considerations, the number of sensors is also a critical metric. In addition to the satisfaction of the requirement on the minimum detection probability thresholds, a second objective in a deployment problem is to minimize the number of sensors. Formally, the aim is to find the best WSN topology to the following multi-objectives optimization problem:

1. Minimize the number of deployed sensors needed to satisfy the following two constraints (2 and 3).
2. For each point *p*_(*i,j*)_ *∈ 𝒜*, minimize the difference between the required detection probability threshold *r*_(*i,j*)_, and the after-deployment resulting detection probability *𝒫*_(*i,j*)_.
3. Ensure the wireless communication network connectivity.

A multi-objectives optimization problem described above is NP-Hard problem. The size of the solution space is finite but very large (2^*n*^2^^). We remind that the deployment field is a square, with a side equals to *n* units. To resolve this optimization problem, we can choose an exact method as Branch & Bound [17] that provides an optimal deployment (solution). Unfortunately, we cannot apply it to large areas because of its exponential complexity. The second alternative is to resolve the problem by using heuristics. Inopportunely, these methods cannot guarantee to obtain the optimal solution. However, the main advantage is the polynomial complexity and the running time which can be reasonable.

In this paper, we propose a pseudo-random algorithm based on Tabu Search heuristic. We resolve our multi-objectives optimization problem on two stages. Firstly, we address our problem by missing the connectivity constraint. We interest only in the two primary objectives: i) minimize the number of sensors, ii) reduce the gap between the generated and requested event detection probabilities. Secondly, we extend our proposal to guarantee the network connectivity.

## 4. Proposal: Tabu Search Approach

Among the possible heuristics able to solve our optimization problem, we choose in this paper the Tabu Search method [18, 19]. This method is a local search optimization technique which tries to minimize a *cost function F* (*x*), where *x* represents a parameter vector, by iteratively moving from a solution *x* to a solution *x′* in the neighborhood of *x* (according to a neighborhood function *H* (*x*)) until a stopping criterion is satisfied or a predetermined number *N* of iterations is reached.

The Tabu Search algorithm is independent from the event detection model. This model provides an input parameters to the method, though some other detections models can be used.

We adapt the Tabu Search algorithm to our Differentiated Sensor Network Deployment problem. The *initialization* of the method, the *neighborhood function*, the *cost function*, and the new specific steps are detailed hereafter.

### 4.1. Resolution without communication connectivity constraint

In this section we present our pseudo-random deployment algorithm based on Tabu Search heuristic. In this stage we do not take into account the communication network connectivity constraint in the deployment process. Connectivity and coverage are related, because they are affected by the sensors position. We assume that a communication range is so large, $R_{c}\geq \sqrt{3}R_{\max}$, in aim to apply the result (necessary conditions to ensure the connectivity) proposed in [12]. Hence, we guarantee the WSN connectivity and we are focused only on the event detection probabilities constraints.

1. ***Initialization***
  The convergence of Tabu Search method depends on the judicious choice of the initial solution (*s*_0_). Ideally, the first solution has to be close to the optimal one, otherwise, since the maximum number of iterations is fixed, the algorithm may stop before reaching the best solution.
  We consider that the decision, *D*(*x, y*), of deploying a sensor in a point *p*_(*x,y*)_ is a random variable, which follows a Bernoulli distribution with parameter *α*_(*x,y*)_. The binary form of the decision rule motivates the choice of the Bernoulli law. Precisely, *D*(*x, y*) can assume a value of 1 with a probability of *α*_(*x,y*)_ and the value of 0 with a probability of (1 *− α*_(*x,y*)_).
  The parameter *α*_(*x,y*)_, associated to a point *p*_(*x,y*)_, is chosen as the percentage of the points located in the vicinity of *p*_(*x,y*)_ and not receiving the required probabilities of detection. The vicinity, denoted *E*_(*x,y*)_, is defined as the set of neighbor points located inside the maximum monitoring circle of a sensor which would be placed in *p*_(*x,y*)_. Formally,
  (3) $$\alpha_{\left(x,y\right)}=\frac{1}{\left\|E_{\left(x,y\right)}\right\|}\sum_{(i,j)\in E_{\left(x,y\right)}}1_{\left\{r_{\left(i,j\right)}>{𝒫}_{\left(i,j\right)}\right\}}$$
  Here 1_{cond}_ is the indicating function, which is equal to 1 if the condition cond is true and 0 otherwise. The initialization stage of our Tabu Search approach follows these steps:
  - **Step 1:** The initial Tabu Search solution is started assuming zero deployed sensors. Thus, ∀*p*_(*x,y*)_ *∈ 𝒜*, Bernoulli parameters are computed using equation 3 with *𝒫*_(*x,y*)_ = 0.
  - **Step 2:** Generate a list, *L_init_*, including all points of *𝒜. L_init_* is a decreasingly sorted list of points according to their Bernoulli parameters.
  - **Step 3:** In *L_init_*, select the point *p*_(*x,y*)_ with the highest Bernoulli parameter, and remove it from the list. If the actual detection probability *𝒫*_(*x,y*)_ associated to *p*_(*x,y*)_ is lower than *r*_(*x,y*)_, then a decision to deploy a sensor in *p*_(*x,y*)_ is randomly generated through a Bernoulli decision rule with parameter *α*_(*x,y*)_.
  - **Step 4:** If the Bernoulli decision is to deploy a sensor (*D*(*x, y*) = 1), then *(1)* the probabilities of detection for all points in the vicinity of *p*_(*x,y*)_ (the set *E*_(*x,y*)_) are recomputed. and *(2) L_init_* is updated (sorted).
  - **Step 5:** If *L_init_* is not empty, go back to **Step 3**.
  When the stop criterion of step 5 is satisfied, the resulting positions of deployed sensors are considered as the initial solution *s*_0_. The latter one is saved in the algorithm’s memory, called the Tabu List. In the remaining, we will refer to this list as *T*. The goal of the Tabu list is not to block the method on a local minimum of the cost function.
2. ***Neighborhood exploration function***
  After the initialization stage, a Tabu Search method executes *N* times the neighborhood exploration stage. Here, *N* is a chosen fixed parameter, which must be set in order to limit the number of Tabu Search iterations and to obtain a satisfactory (near to the optimal) solution.
  During the *n^th^* iteration of the neighborhood exploration stage, a given number *V* of possible neighbors of the solution selected in the previous iteration, noted *s*_*n*−1_, are generated and evaluated. Neighboring solutions are possible solutions which can be reached from *s*_*n*−1_ by a basic transformation. Solutions which are present in the Tabu List *T* are considered unreachable neighbors.
  We propose two neighboring generation methods, namely **Suppression oriented stage** (*H_supp_*) and **Additional oriented stage** (*H_add_*). These two methods alternate in the successive iterations of our Tabu Search approach in order to determine the set *V*. Both stages are detailed hereafter.
  - ***Suppression oriented stage*** (H*_supp_*):
    The aim of this stage is to suppress some sensors among those deployed in over-covered areas. The method proceeds with the following steps:
    – **Step 1’:** Compute the Bernoulli parameters for all *p*_(*x,y*)_ *∈ 𝒜* using the equation 4 and assuming the deployment obtained in the last Tabu Search iteration.
      (4) $$\beta_{\left(x,y\right)}=\frac{1}{\left\|E_{\left(x,y\right)}\right\|}\sum_{(i,j)\in E_{\left(x,y\right)}}\left[(1-\frac{r_{\left(i,j\right)}}{{𝒫}_{\left(i,j\right)}})\times 1_{\left\{r_{\left(i,j\right)}<{𝒫}_{\left(i,j\right)}\right\}}\right]$$
    **– Step 2’:** Generate a list, *L_supp_*, including all the points of *𝒜* where a sensor is deployed (*D*(*x, y*) = 1). The list is then decreasingly sorted according to the resulting Bernoulli parameters.
    **– Step 3’:** In *L_supp_*, select and remove the point *p*_(*x,y*)_ with the highest Bernoulli parameter, and randomly generated the decision to suppress the sensor in *p*_(*x,y*)_ through a Bernoulli decision rule with parameter *β*_(*x,y*)_.
    – **Step 4’:** If the Bernoulli decision is to suppress the sensor (*D*(*x, y*) = 0), then the probabilities of detection for all points in the vicinity of *p*_(*x,y*)_ are recomputed. In this case, the list *L_supp_* is updated (sorted) with the new values of the Bernoulli parameters associated to each point in *L_supp_*.
    – **Step 5’:** If *L_supp_* is not empty, go back to **Step 3’**.
    Once the stop criteria on step 5’ is satisfied, the next Tabu Search iteration alternates toward an additional stage.
  - ***Additional oriented stage*** (H_add_):
    The aim of this stage is to add more sensors to the actual deployed ones in under-covered areas. The execution is very similar to the initialization stage, except Step 1 and Step 2 are replaced by the following steps:
    – **Step 1”:** Compute the Bernoulli parameters for all *p*_(*x,y*)_ *∈ 𝒜* using the equation 3 and assuming the deployment obtained in the last Tabu Search iteration.
    – **Step 2”:** Generate a list *L_add_* including all the points of *𝒜* where a sensor is not deployed. The list one is then decreasingly sorted according to the resulting Bernoulli parameters.
    Steps 3, 4 and 5, as detailed in initialization stage, are repeatedly executed until that the list *L_add_* is empty.
3. ***Cost function and selection of new solution***
  After the neighborhood exploration (a suppression or an additional oriented stage) during the *n^th^* iteration, an elected solution *s_n_* must be chosen among the *V* explored candidates. This solution (which cannot be in the Tabu list *T*) is the one which provided by minimizing a given cost function *F*. The cost function reflects two objectives of the optimization problem described in section 3.2., minimize the number of deployed sensors and reduce the gap between the generated and requested event detection probabilities. The first objective could be quantified by counting the number of deployed sensors. Formally, as defined in section *D*(*x, y*) = 1, if a sensor is deployed in the point *p*_(*x,y*)_. Otherwise, *D*(*x, y*) = 0. In order to minimize the cost function, *F* includes the following term:
  (5) $$\sum_{(x,y)\in 𝒜}D(x,y)$$
  The second objective is integrated into the cost function through the following *penalty function*:
  (6) $$\mathrm{Penalty}=\sum_{(x,y)\in 𝒜}\frac{{\left[r_{\left(x,y\right)}-{𝒫}_{\left(x,y\right)}\right]}^{+}}{r_{\left(x,y\right)}}$$
  Here [*r*_(*x,y*)_ *− 𝒫*_(*x,y*)_]^+^ denotes the projection of *r*_(*x,y*)_ *− 𝒫*_(*x,y*)_ in *IR* ^+^. Formally,
  (7) $${\left[r_{\left(x,y\right)}-{𝒫}_{\left(x,y\right)}\right]}^{+}=\left(r_{\left(x,y\right)}-{𝒫}_{\left(x,y\right)}\right)\times 1_{\left\{r_{\left(x,y\right)}>{𝒫}_{\left(x,y\right)}\right\}}$$
  According to the expression of penalty function, a successful deployment solution should lead to obtain detection probabilities higher than (or ideally equal to) the required detection thresholds. If it is not satisfied, the penalty function value translates how far is the solution to the required thresholds. This is exactly the second objective of the optimization problem.
  From the above objective expressions, the authors define two cost functions *F_supp_* and *F_add_*. The function *F_supp_* is used to choose the best next iteration solution in the case of a *suppression oriented stage*. The function *F_supp_* is formulated using only the equation 6. On the other hand, the cost function *F_add_* is used in the case of *additional oriented stage*. In this case, the two both terms associated to each objective of optimization problem are integrated into the cost function through the following additive expression:
  (8) $$F_{\mathrm{add}}=\sum_{(x,y)\in 𝒜}[D(x,y)]+\mathrm{Penalty}$$

The pseudo code of Tabu search deployment process is illustrated in Algorithm 1.

**Algorithm 1:**
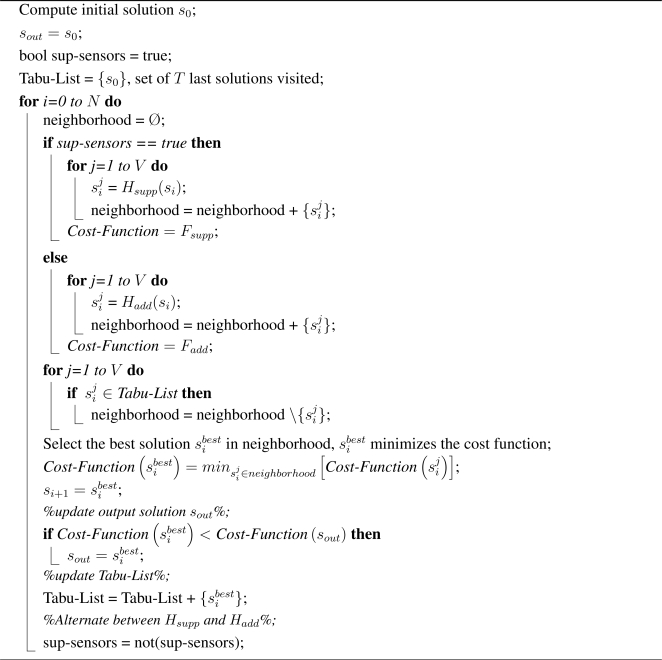
*Tabu search* pseudo code

The computational complexity of Tabu search is equal to *O*(*NV m*^2^*n*^2^). *N* is the number of iterations of Tabu search process and *V* is the size of the neighborhood. These two parameters are chosen by the designer, they are calibrated according to the specific deployment scenario. A coverage circle of sensor covers a set of cells in the area, *m* represents the number of cells in length or width of sub-area covered by a sensor. *m* depends on the maximum radius circle *R_max_*, *m* is equal to $\left\lceil \frac{R_{\max}}{\sqrt{2}}\right\rceil$. We remark that the Tabu search complexity depends on the deployment area dimensions, detection characteristic of sensor (*R_max_*), and parameters of Tabu Search algorithm (*N*, *V*). If *N*, *V* and *m* are not large, the product *NV m*^2^ is equal to constant “a”, so we have a quadratic complexity *O*(*a.n*^2^).

### 4.2. Extension: Wireless Communication Network Connectivity

In this section, we present the extended version of Tabu Search deployment presented above. The extension consists to guarantee the communication network connectivity for any communication range value, so all constraints of optimization problem (minimize number of sensors, reduce the gap between the generated and requested event detection probabilities, network connectivity) are included formally in our proposal. We adapt the initialization stage and the selection process of solutions in the neighborhood to build the network connectivity. The remainder steps and cost functions are identical to those presented in section 4.1.. Hereafter, we detail the modifications made to the initialization stage and the selection of new solution process.

1. ***Initialization stage***
  Compared to the initialization stage described in section 4.1., the new version differs in the fact that once the Bernoulli decision to deploy the first sensor is made, the only points which will be tested (i.e. Bernoulli decision to deploy or not to deploy a sensor) are those which are located inside the communication range *R_c_* of any deployed sensor. The connectivity is thus guaranteed by construction (gradually).
  Moreover, the selection order of the cell to be tested follows a decreasing order of the difference between the requested and the generated detection probabilities. That is, the next tested point is *p*_(*i,j*)_ = max_*p*_(*x,y*)_∈*𝒝*_ (*𝒫*_(*x,y*)_ − *r*_(*x,y*)_), where *𝒝* denotes the set of points of *𝒜* which have not been tested yet at this stage.
  The positions of the resulting deployed sensors are considered as the initial solution *s*_0_. The latter one is saved in the algorithm’s memory (Tabu List).
2. ***Selection of new solution***
  After the neighborhood exploration (a suppression or an addition oriented stage) during the *n^th^* iteration, an elected solution *s_n_* must be chosen among the *V* (size of the neighborhood) explored candidates. For each candidate, say *C*, we construct the graph of connectivity, denoted *G*(*V, E*). Here, *V* is the vertices set. The latter one is exclusively composed of points where, according to solution *C*, a sensor will be deployed. Moreover, *E* is the edges set. Formally,
  (9) $$E=\{\forall (p_{\left(x_{1},y_{1}\right)},\;p_{\left(x_{2},y_{2}\right)})\in V|p_{\left(x_{1},y_{1}\right)}\neq p_{\left(x_{2},y_{2}\right)}\;\;\text{and}\;\;\left\|p_{\left(x_{1},y_{1}\right)}\;p_{\left(x_{2},y_{2}\right)}\right\|\leq R_{c}\}$$
  In the *V* solutions generated by the suppression stage, we eliminate the ones where the resulting communication graph *G* is not connected. Among the remaining candidates, the elected solution, *s_n_*, is the one which minimizes a given cost function *F*.

## 5. Performance Analysis

In order to evaluate and to compare the performance of our Tabu Search approach deployment method, we implemented our proposal and the deployment strategies found in the literature, namely: Random, Grid, Min_Miss [9], Max_Min_Cov [8], Max_Avg_Cov [8], Diff–Deploy [10], and DAA [11].

The comparison is based on different metrics as number of sensors deployed, satisfaction rate in detection probabilities *θ* (percentage of area units receiving a detection probability larger or equal than the requested event detection probability threshold), computational complexity, memory consumption, and network communication connectivity. We compare the deployment methods in two stages. Initially, we focus the comparison on all the metrics introduced bellow except network connectivity. Afterwards, the comparison will include the network connectivity.

**Figure 2.**
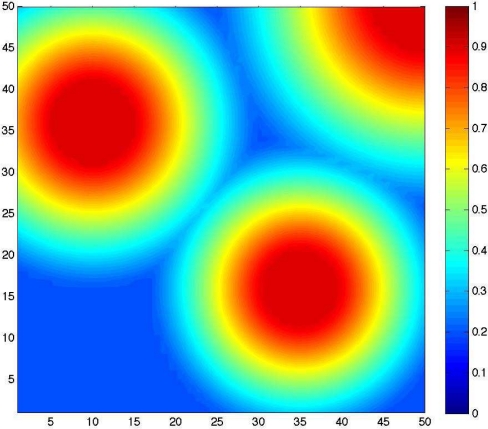
Area 50*50 with its desired detection probability values

We fixed sensor parameters values, *α*, *β*, *R_max_*, and *R_c_* to 1, 1, 5, and 3 respectively. We chose *R_c_* less than $\sqrt{3}R_{\max}$ in order to illustrate how our deployment algorithm can ensure and build the connectivity with a small connectivity range. We remainder that if *R_c_* is greater than $\sqrt{3}R_{\max}$, the sufficient condition to guarantee the connectivity is the full coverage of deployment area. We considered an area with 50 * 50 units. The required detection probabilities thresholds are illustrated in Figure 2, they vary from 0.2 to 0.9.

For a regular deployment we chose a grid topology, so the shape is rectangle. In Mesh method, we chose the triangle shape for meshes. To divide a mesh, we placed a new sensor in the middle of one given arc. The cost function is the miss detection probabilities rate, it is equal to (1 − *θ*).

We calibrated the Tabu search process by fixing the number of iterations, the size of the Tabu list and the size of neighborhood explored to 100, 10, and 15 respectively. We fixed a confidence level for results of stochastic deployment strategies (Tabu Search and Random) to 99.70%.

**Figure 3.**
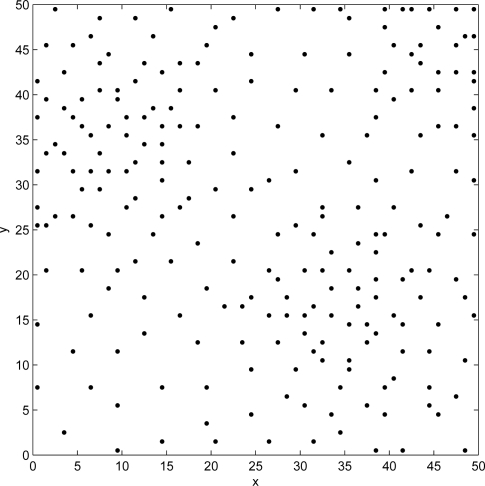
Tabu Search approach: Sensors positions

Figure 3 shows the deployed sensors positions obtained by our Tabu Search approach. The number of deployed sensors is equal to 234 ± 2 sensors, for a satisfaction rate *θ* is equal to 97.10 ± 0.24%. For the same number of sensors, 234, the satisfaction rate obtained when using the Random, Grid, Min_Miss, Max_Min_Cov, Max_Avg_Cov, Diff–Deploy, and DDA approaches are equal to 73.45± 1.06%, 83.12%, 85.88%, 83.36%, 82.56%, 96.72%, and 93.24% respectively.

However, to reach the satisfaction rate obtained by the Tabu Search approach (97.10%), in table ?? we show the minimal number of sensors that above deployment strategies must deploy.

We can notice from the above results that our proposal Tabu Search and Diff–Deploy reduce highly the number of deployed sensors while improving the satisfaction rate. But the computational complexity of Diff–Deploy is equal to $O\left(\frac{4}{3}n^{6}\right)$ is larger than the computational complexity of our proposal Tabu Search which is equal to *O*(*a.n*^2^). Also the memory consumption in Diff–Deploy is more important than the memory consumed in Tabu Search, because Diff–Deploy manipulates square matrix with *n*^4^ elements. DDA method gives also a satisfactory result but is more complex (*O*(*n*^6^)) than Tabu Search. Hence, we conclude that our proposal is more scalable than the deployment strategies found in the literature.

In Figure 3, we plot the sensors positions using the Tabu Search approach. Compared to Figure 2 we can notice a clear concentration of the deployed sensors in the areas requiring high detection probability thresholds (top right, top left, and down right).

**Figure 4.**
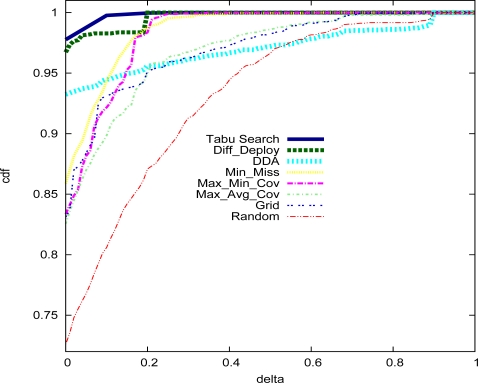
cdf between desired and obtained detection probability

Figure 4 illustrates the cumulative distribution function (cdf) obtained by all the deployment strategies of *X*, where *X* is a random variable, which can take values in the set of {*ϑ_i,j_*|∀*i, j* ∈ *𝒜*}. Formally,

(10) $$\vartheta_{i,j}=(r_{\left(i,j\right)}-p_{\left(i,j\right)})\times 1_{\left\{r_{\left(i,j\right)}>p_{\left(i,j\right)}\right\}}$$

*ϑ_i_* can take values in [0, 1]. Each curve in Figure 4 indicates for each *δ* value in the *x* axis the probability *P* (*X* ≤ *δ*). From the under figure, we can notice that our proposal Tabu Search, Diff–Deploy and DDA approaches provide the best performances compared to all other deployment algorithms. Moreover, we can observe that the Tabu Search approach satisfaction rate reaches very quickly 100% of the area units compared to the other approaches. This means that the not satisfied units receive a detection probability very close to the required detection probability thresholds.

**Figure 5.**
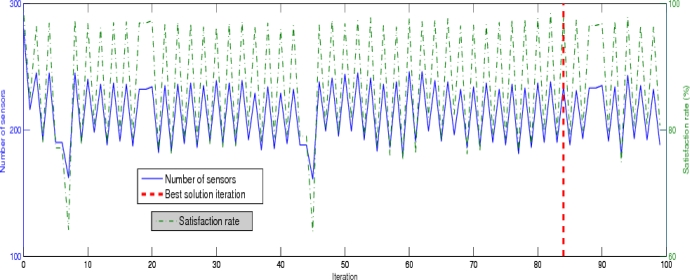
Tabu Search: evolution of iterations

In figure 5, we illustrate the variation of the satisfaction rate and the number of deployed sensors at each Tabu Search iteration. A left y-axis represents the number of deployed sensors and a right y-axis shows the satisfaction rate. We remark that the number of sensors and the satisfaction rate oscillate, this behavior is due to the additional and suppression oriented stages. When we execute the suppression stage, the number of sensors is largely reduced consequently the satisfaction rate also is decreased. The suppression stage enables to the additional stage to generate different WSN topologies compared to the last one. Thereafter, we apply the additional stage in aim to deploy more sensors in areas under-covered. We remind that our aim is to minimize the number of deployed sensors with an additional stage and to reduce the gap between the generated and requested event detection probabilities. We can see in figure 5 that the number of sensors placed by the additional oriented stage is declining but the look of the satisfaction rate plot is constant (no variation). Besides, sometimes we have a growth curve of number of sensors. The main reason is the Tabu Search heuristic, it allows an impoverishment in the performance in order to do not be blocked in the local minima. The best solution among 100 Tabu Search iterations is obtained at the 84*^th^* iteration, in figure 5 is represented with a red vertical line.

**Figure 6.**
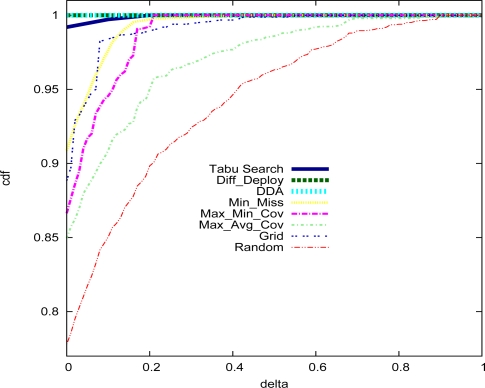
cdf between desired and obtained detection probability considering connectivity

In order to compare the network connectivity between the different approaches, we run the second version of Tabu Search where the connectivity is ensured by construction. Tabu Search method deploys 274 ± 2 sensors and the satisfaction rate *θ* is equal to 99.01 ± 0.12%. With the same number of sensors (274), the satisfaction rate *θ* of Random, Grid, Min_Miss, Max_Min_Cov, Max_Avg_Cov, Diff–Deploy, and DDA approaches are equal to 79.51 ± 1.02%, 88.84%, 90.88%, 86.64%, 84.96%, 100% and 100% respectively. We can observe that Diff–Deploy and DDA reach 100% in satisfaction rate. Figure 6 shows the cumulative distribution function (cdf) of random variable X defined in equation 10.

**Figure 7.**
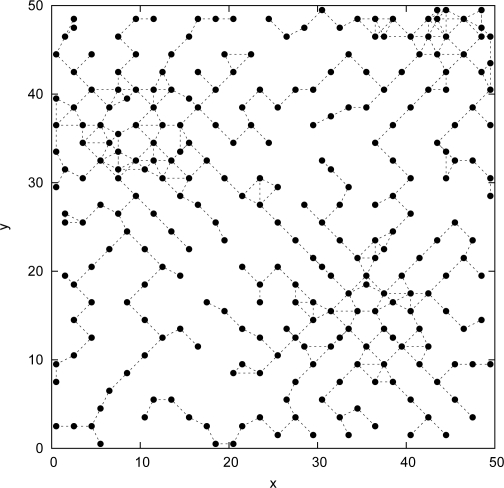
Tabu Search: Wireless communication network graph, *R_c_* = 3

**Figure 8.**
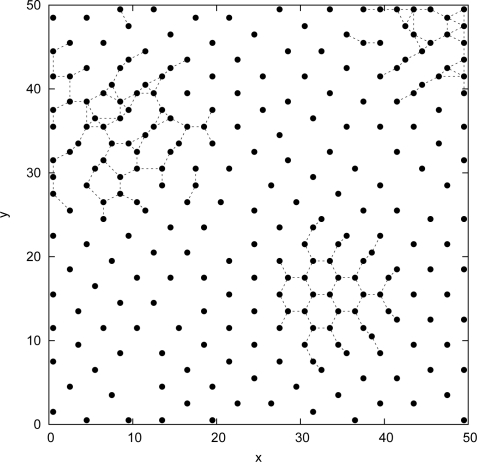
Diff-Deploy: Wireless communication network graph, *R_c_* = 3

We calculate the number of connected components in all deployment topologies generated by the different deployment strategies. The network is connected if the graph of connectivity contains only one connected component. Figures 7 and 8 illustrate the graph of connectivity of Tabu Search and Diff–Deploy. We can see clearly that only our proposal Tabu Search approach provides a connected graph.

**Figure 9.**
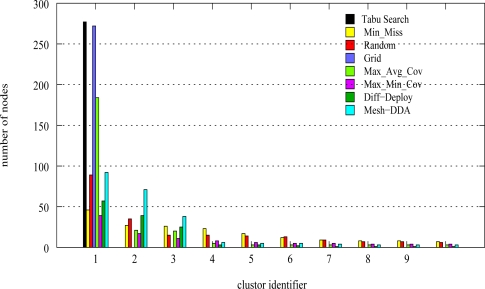
Connected components of wireless network communication graphs

Finally, in figure 9 we illustrate the connected components related to the connectivity graphs resulting from the various deployment methods. We represent the number of nodes for each connected component. We plot only the 10 largest connected components related to each graph. A largest connected component is the one which contains the greatest number of nodes. We note that only the graphs resulting from the Tabu Search approach and the Grid have one connected component. In the case of the Grid, we certainly guarantee the connectivity but the performances in term of detection probabilities are not satisfactory (see Figure 6). The number of sensors deployed in the Grid is slightly lesser than that Tabu Search. The cause is due to the construction of the grid shape. The other methods contain more than one connected component. Which means that the wireless communication network is not connected. For example Diff–Deploy and DDA have 134 and 41 connected components respectively. Tabu Search method is the only method which ensures the connectivity and produces a better satisfaction rate compared to other methods, while minimizing the number of required sensors.

## 6. Conclusion and Future Work

Assuming both a probabilistic detection model and the geographical irregularity of the sensed events, we propose in this paper a simple and scalable pseudo-random deployment approach for WSN which guarantees the wireless network communication connectivity. Our proposal is based on the Tabu Search heuristic. The obtained results show that our proposal achieves a much better satisfaction rate than several other approaches proposed in the literature ( Random, Grid, Min_Miss, Max_Min_Cov, Max_Avg_Cov, Diff–Deploy, and DDA).

In our ongoing work, we are studying the MAC layer impact on the network lifetime as new constraint in the deployment process. Our objective is to maximize the network lifetime (reduce the energy consumption). We focus our work to model IEEE 802.15.4 and to adapt the positions and the number of sensors according to the MAC layer specificities.

## Figures and Tables

**Table 1.** Number of deployed sensors to reach 97.10% of satisfaction rate

Deployment strategy	Number of sensors
Random	570
Grid	540
Min_Miss	351
Max_Min_Cov	385
Max_Avg_Cov	524
Diff–Deploy	235
DDA	251
Tabu Search	234

## References

[1] Akyildiz I.F., W. Su Y. S., Cayirci E. (2002). Wireless Sensor Networks: a Survey. Computer Networks: The International Journal of Computer and Telecommunications Networking.

[2] Huang C.-F., Tseng Y.-C. (2003). The Coverage Problem in a Wireless Sensor Network.

[3] Correia L. H. A., Macedo D. F., dos Santos A.L., Loureiro A.A.F., Nogueira J.M.S. (2007). Transmission Power Control Techniques for Wireless Sensor Networks. Elsevier Computer Networks.

[4] Maleki M., Pedram M. (2005). Qom and Lifetime-constrained Random Deployment of Sensor Networks for Minimum Energy Consumption.

[5] Chan H., Perrig A., Song D. (2003). Random Key Predistribution Schemes for Sensor Networks.

[6] Kar K., Banerjee S. (2003). Node Placement for Connected Coverage in Sensor Networks.

[7] Chakrabarty K., Iyengar S.S., Qi H., Cho E. (2002). Grid Coverage for Surveillance and Target Location in Distributed Sensor Networks. IEEE Trans. Comput.

[8] Dhillon S., Chakrabarty K., Iyengar S. (2002). Sensor Placement for Grid Coverage under Imprecise Detections.

[9] Zou Y., Chakrabarty K. (2004). Uncertainty-Aware and Coverage-Oriented Deployment for Sensor Networks. J. Parallel Distrib. Comput.

[10] Zhang J., Tan T., S.H S. (2006). Deployment Strategies for Differentiated Detection in Wireless Sensor Networks. SECON’06.

[11] Aitsaadi N., Achir N., Boussetta K., Pujolle G. (2007). Differentiated Underwater Sensor Network Deployment. IEEE/OES OCEANS’07.

[12] Bai X., Kuma S., Xua D., Z. Y., Lai T. (2006). Deploying Wireless Sensors to Achieve Both Coverage and Connectivity.

[13] Y. Wang C. H., Tseng Y. (2005). Efficient Deployment Algorithms for Ensuring Coverage and Connectivity of Wireless Sensor Networks.

[14] Elfes A., Iyengar S. S., Elfes A. (1991). Occupancy Grids: A Stochastic Spatial Representation for Active Robot Perception. Autonomous Mobile Robots: Perception, Mapping, and Navigation (Vol. 1).

[15] Kuo S.-P., Tseng Y.-C., Wu F.-J., Lin C.-Y. (2005). A Probabilistic Signal-Strength-Based Evaluation Methodology for Sensor Network Deployment.

[16] Li X., Wan P., Wang Y., Frieder O. (2002). Coverage in Wireless Ad-hoc Sensor Networks.

[17] Brusco M., Stahl S. (2005). Branch-and-Bound Applications in Combinatorial Data Analysis.

[18] Glover F. (1989). Tabu Search - Part I. ORSA journal on Computing.

[19] Glover F. (1990). Tabu Search - Part II. ORSA journal on Computing.

